# Celebrating year of the nurse and the midwife: time to tell your stories

**DOI:** 10.4069/kjwhn.2020.05.14

**Published:** 2020-06-11

**Authors:** Emily E. Drake

**Affiliations:** School of Nursing, University of Virginia, Charlottesville, VA, USA

**Keywords:** Art, History, Nursing, Pandemics, World Health Organization

I have never been more proud to be a nurse. In May 2019 when the World Health Organization (WHO) declared that 2020 would be “The Year of the Nurse and Midwife” at the 72nd World Health Assembly [1] they could not have known that 2020 would also become the year of the coronavirus disease (COVID-19), the novel coronavirus pandemic. Their goal was to empower nurses and to emphasize the need for more nurses and midwives worldwide. The WHO has highlighted the stories of nurses around the world: conducing cervical cancer screening in England, providing vaccines in Guatemala, caring for homeless people in Ireland, and making childbirth safer in the Philippines [2]. Their goal was to shine a light on the vital role that nurses play in health care now and in the future.

Now during the COVID-19 pandemic, people under quarantine call us heroes and clap from their windows and apartment buildings as we go into the hospital each shift. However, it is important to note that nurses are not just in the hospital, they’re also in clinics, out in the community, conducting research, working on policy, and serving in leadership roles. We are taking care of patients as well as contributing to decisions on how best to handle this pandemic on a community level. Our nursing expertise applies in many settings, and our reach can extend from the cellular level to society.

The International Council of Nurses calls for photos or short films of nurses in action to commemorate the Year of the Nurse and Midwife [3]. Sigma, the International Nursing Honor Society, encourages sharing the creative work of nurses and helps to publish that work [4]. The Korean Nurses Association is posting photos and stories documenting the pandemic [5]. As another example of an online photo-voice project, Adelene Egan, a nurse in New York City wanted to uplift her coworkers through photography and storytelling and she created #Faces of the Frontlines https://addieegan.wixsite.com/mysite. Historians are asking us to document this pandemic, and nurses can help.

According to the Woodhull Study Revisited [6] nurses are rarely interviewed or quoted for public news stories about health. Are we invisible and unheard, or are we not putting our voice out there? During this pandemic, we are heroes and never before has the public so appreciated what we do. Now is the time for us to tell our stories!

There is power in story. By telling our story, highlighting our work and our profession, we are empowering our peers, ourselves, and our patients. These stories can be therapeutic for the storyteller and powerful to the receiver. Stories can inspire us and challenge us to think in new ways. Stories can tap into the core values of our work. Stories help to preserve culture, to document knowledge, and to stimulate change.

Storytelling represents a different way of learning about the world. There are multiple ways of knowing through art, poetry, and humor, as well as empirical research. Stories of direct experience serve as a valuable adjunct to research and can be used to highlight research findings or to provide ideas for future research projects. It’s important to share lessons learned from this pandemic that can help us as a profession and that can help advance science.

Negussie [7] suggests that we need to gather stories of cultural wisdom and traditional, inherited knowledge and that we need to respect and appreciate them along with modern scientific findings. Anthropologists and ethnographers have long known the value of collecting stories from different cultures. Preserving and sharing these stories can help us think globally. We can begin to embrace a multicultural perspective through the telling of each other's stories.

Storytelling reflects Belenky’s theory of women’s ways of knowing [8] and Carper’s esthetic, personal, and ethical ways of knowing [9]. Exemplars are stories that include an interpretive component [10]. An exemplar can describe scenarios that are unusual, as well as those that are ordinary or typical. A good exemplar captures the essence of an experience through rich description that includes context and reflects the storyteller's concerns, thoughts, and feelings. These stories and exemplars are useful in that they make explicit the knowledge gained from our daily experiences, and the act of sharing them helps build communities of practice.

Business leaders and consultants often use stories to transform corporate organizations. Storytelling can be a powerful tool for catalyzing change. Sharing stories builds trust. Complex ideas can be conveyed in a meaningful way that helps people focus on a new idea in a nonthreatening manner. Each person who hears the story begins to co-create new stories and to think about solutions. Stories make us challenge our old ways of thinking and look critically at our traditional practices. Stories can provide a vision of what is past and what is possible. Telling the story can also transform the teller.

Good story telling can also be challenging. Nurses work hard to earn the trust of their patients and value confidentiality, so we are great secret keepers. Nurses may be concerned about how their patients or employers will view them if they tell their stories publicly. There must be a way to tell stories so that nurses’ work is made visible and expertise is made known. By removing or changing the patient’s name, by leaving the location vague, nurses can still tell their stories. And if not your story, tell the story of other nurses. Tell a story about a nurse or colleague that you admire and what you have witnessed in action.

Nurses need to tell these stories, not just to each other but, more importantly, to others. Tell these stories to administrators, other health care professionals, government officials, lay people, friends and family. Tell them what we do as nurses. Tell them what we see. Nurses are witness to the grand human experience and have a unique perspective, especially during this pandemic.

## A call to action

Nurses and readers of *Korean Journal of Women Health Nursing* (KJWHN). Now is the time to tell your story. If you can’t tell a long story, tell it in a tweet, just one or two sentences. If you don’t feel comfortable sharing a story, then share a piece of art, a poem, a drawing—something that illustrates what we as nurses do. Paint a picture. Write a poem. Take a photo. Writing in a journal, sharing a story, or creating a work of art is good for you, it’s good for others, and it’s good for the profession. Art has been used as a tool to teach nursing and medical students [11]. By telling your story you can help inspire others. You can also help recruit others to our field. We need more nurses. We especially need more nurses caring for women and families.

We can help give voice to our patients and their needs. For the sake of your patients, tell their story. Share their bravery, their everyday struggles and the specific challenges they face. Tell stories that illustrate what they need. Be their advocate. Be a champion for them. Speak for those who can’t speak for themselves. Tell your story not just for you, but in order to help others.

What do nurses do every day? We care for human beings, in a holistic way, the person and environment. We care about harmony and balance. We treat not just the disease, but also the human response to health and illness. We are involved in health promotion, disease prevention, symptom management, and alleviating suffering. We are also the guardians of safety, champions of quality improvement, and are expert problem-solvers. Nurses care about public health, climate change, infectious disease, immunizations, emergencies and disasters. We advocate for patients from birth to death; we deliver babies and we provide compassionate end-of-life care. From within the pages of KJWHN you can read the stories of how nurses research, educate, innovate, and advocate for the care of childbearing families, and advance the care of infertility, preterm labor, sexuality, breast and ovarian cancer, lesbian gay bisexual transgender and queer (LGBTQ) persons, human papillomavirus vaccination, depression, sleep, obesity, aging, and family centered care.

Start each day with a grateful heart. Nurses are heroes. The future of health care is in our hands. As the WHO intended, this is our year! Please tell your story.
